# Efficacy of Tranexamic Acid in Reducing Myomectomy-Associated Blood Loss among Patients with Uterine Myomas at Federal Teaching Hospital Abakaliki: A Randomized Control Trial

**DOI:** 10.1155/2024/2794052

**Published:** 2024-01-18

**Authors:** Ayodele Adegbite Olaleye, Joshua Adeniyi Adebayo, Justus Ndulue Eze, Leonard Ogbonna Ajah, Chidebe Christian Anikwe, John O. Egede, Chidi Ikenna Ebere

**Affiliations:** ^1^Department of Obstetrics and Gynaecology, Alex Ekwueme Federal University Teaching Hospital, Abakaliki, Nigeria; ^2^Department of Obstetrics and Gynaecology, University of Nigeria Teaching Hospital, Enugu, Nigeria; ^3^Department of Obstetrics and Gynaecology, Nnamdi Azikiwe University Teaching Hospital, Nnewi, Nigeria

## Abstract

**Background:**

Myomectomy can be associated with life-threatening conditions such as bleeding. Excessive bleeding usually necessitates blood transfusion. Interventions to reduce bleeding during myomectomy will help reduce the need for blood transfusion with its associated complications. Tranexamic acid has been used to reduce bleeding in other surgical procedures, and its usage during myomectomy merits evaluation.

**Objective:**

To assess the efficacy of tranexamic acid in reducing myomectomy-associated blood loss.

**Materials and Methods:**

This is a prospective double-blinded randomized trial conducted on women who had abdominal myomectomy. Patients were randomized into two groups. The study group received perioperative intravenous tranexamic acid (TXA) while the control group received a placebo. Intraoperative blood loss was calculated by measuring the volume in the suction apparatus and weighing the surgical swabs. In addition, blood collected postoperatively from the wound drains and drapes were measured. Haemoglobin concentrations were determined preoperatively and on second postoperative day for all cases. Any adverse effect was noted in both groups. The data was processed using Epi Info software (7.2.1, CDC, Atlanta, Georgia). The relationships between categorical data were analyzed using *X*^2^ and Student's *t*-test to determine relationships between continuous variables, with a *P* value of 0.05 considered statistically significant, and correlation coefficients were calculated using Pearson's formula, and probability of 0.05 was set for statistical significance.

**Results:**

Symptomatic uterine myomas constituted 17.3% of all gynaecological admissions and 21.3% of gynaecological operations at Federal Teaching Hospital Abakaliki. The mean intraoperative blood loss among patients that had perioperative tranexamic acid infusion was 413.6 ± 165.6 ml, while that of patients with placebo infusion was 713.6 ± 236.3 ml. Perioperative tranexamic acid infusion therefore reduced mean intraoperative blood loss by 300 ml, and this was statistically significant (SMD = −0.212, 95% CI: −403.932 to −196.067, *P* < 0.0001). Perioperative tranexamic acid reduced mean total blood loss by a value of 532.3 ml, and this is statistically significant (SMD = 30.622, 95% CI: 393.308 to 670.624, *P* < 0.0001). Tranexamic acid also improved postoperative haemoglobin concentration by 1.8 g/dl compared with placebo, and this is statistically significant (SMD = −0.122, 95% CI: 1.182 to 2.473, *P* < 0.0001). Tranexamic acid infusion decreased hospital stay by about 2 days, and this difference was statistically significant (SMD = −3.929, 95% CI: -3.018 to –0.983, *P* = 0.0003). There was no adverse drug reaction in the course of the study.

**Conclusion:**

The use of tranexamic acid during myomectomy reduced intraoperative and postoperative blood loss. It is also associated with decreased hospital stay. This trial is registered with NCT04560465.

## 1. Introduction

Uterine fibroids are the most common benign gynaecological tumours of the female genital tract [1–3]. The true incidence is unknown as majority of cases are asymptomatic; however, evidence from symptomatic patients shows that about 20% to 30% of women would be diagnosed at a time during their lives [1]. Fibroids can be located at different sites in the uterus and sometimes protrude into the inner wall of the uterine cavity [2, 3]. The common symptoms are abdominal pain, menorrhagia, and symptomatic anaemia [1–3]. It may also have an impact on fertility when it is located at the submucosal level or blocks the entrance to the fallopian tubes [2, 3].

Various methods are available for the treatment of symptomatic fibroids, and these include medical, surgical, and radiological interventions [4, 5]. Myomectomy remains the most common surgical method for those who have fibroids and desire further childbearing or just want to preserve their uterus [4]. However, substantial perioperative blood loss has been associated with this surgical procedure; and sometimes hysterectomy has to be performed to control bleeding, with attendant increased morbidity and mortality [5].

Though many strategies have been used to reduce blood loss during myomectomy, which include mechanical tourniquet application, administration of hemostatic agents (e.g., fibrinogens and vitamin K), autologous donation, and minimally invasive procedures [6–9], however, blood transfusions are still required to treat anaemia in many cases. Due to the risks associated with allogenic blood transfusion, such as viral infections, immunologically mediated diseases, and cardiovascular dysfunction, as well as the increased financial burden on patients, it is important to continue to find means of reducing myomectomy-associated blood loss in order to circumvent these problems.

Tranexamic acid (TXA) has become popular in reducing blood loss in a number of surgical procedures such as orthopedics, cardiac surgery, general surgery, gynaecologic, and obstetric procedures, as well as during organ transplant surgeries [10–13]. In gynaecology, TXA has been widely used clinically to stop heavy menstrual bleeding. Systematic reviews of randomized control trials (RCTs) including over 25,000 patients that used tranexamic acid in elective surgery showed that it reduced the risk of blood transfusion by 34% without an increased risk in venous thromboembolism (VTE), a known side effect of the drug, or other adverse perioperative outcomes [13].

Tranexamic acid (TXA) is a synthetic analogue of an amino acid lysine, whose biological activity inhibits plasminogen from dissolving clots [9]. With its antifibrinolytic effects, it inhibits both plasminogen activation and plasmin activity, thus preventing clot breakdown rather than promoting new clot formation [9]. Its onset of action is 5-15 minutes, and it remains effective for up to 3 hours. It binds primarily to plasminogens, and about 3% is bond to plasma protein in the circulation. The half-life is 2-11 hours, and it is excreted unchanged in the urine. The common side effects include seizure, ocular impairments, renal impairments, and thromboembolism. It is generally well tolerated in well-selected cases, though contraindicated in patients with acquired defective colour vision, active intravascular clotting, and hypersensitivity to TXA [9, 11].

Despite its documented effects on blood loss in different surgical procedures, TXA acid is not yet part of the protocols for myomectomy in many centres. In a randomized controlled trial, 10 mg/kg of intravenous TXA was found to be effective in reducing blood loss during myomectomy. However, further studies were suggested to affirm this finding [13]. In another RCT comprising eight arms and 571 patients (TXA = 304 patients, control = 267 patients), TXA was found to reduce the mean intraoperative blood loss by average value of 224.34 ml (95% CI [-303.06, -145.61], *P* < 0.001), and mean postoperative blood loss and mean total blood loss were significantly reduced in favor of the prophylactic TXA [14].

In view of the limited numbers of RCTs conducted on efficacy of TXA vs. placebo, additional research on the topic of utmost importance to consolidate the evidence from different populations is needed. Also, considering the need to reduce blood loss during myomectomy and the need for further work on tranexamic acid in that regard, this study was conceived.

## 2. Null Hypothesis

There is no difference in the total blood loss among patients that had tranexamic acid compared with placebo.

## 3. Alternate Hypothesis

Tranexamic acid is superior to placebo in reduction of blood loss during myomectomy.

## 4. Study Design

This was a double-blind randomized controlled trial of efficacy of TXA in reducing myomectomy-associated blood loss at Alex Ekwueme Federal University Teaching Hospital, Abakaliki, Ebonyi State. The open myomectomy procedure and the study protocol and aim were explained to the participants, and written informed consent was obtained. Eligible patients scheduled for open (laparotomic) myomectomy, who met the inclusion criteria, were randomized into two groups (A and B). Parenteral TXA 1000 mg was administered to participants in group A and placebo (water for injection) to participants in group B. The drug or placebo was added into 1000 ml of normal saline and allowed to drop at the rate of 100 ml per hour during the course of the surgery.

The minimum sample size was determined using statistical formula for randomized control trial for superiority study design. This was set at 80% power to detect 220 ml of difference in blood loss as extrapolated from previous studies, with significant level of 5% and a one-sided alpha of 0.05 and beta (*β*) set at 0.1.

(1) $$\begin{array}{c} N=\frac{2\sigma^{2}\left\{Z_{1-\alpha /2}+Z_{1-\beta }\right\}}{{▲}^{2}},\\ N=\frac{2{\left(284\right)}^{2}{\left(0.84+1.96\right)}^{2}}{{\left(220\right)}^{2}},\\ N=26.13\approx 26. \end{array}$$

Adding 20% attrition, *N* = 26 + 5.2 = 31.2 ≈ 31 (for one group). For both groups,*N* = 31 × 2 = 62.

### 4.1. Patients' Selection

Most cases of uterine myomas were diagnosed at gynaecological clinic while some, especially those with menorrhagia, were admitted through gynaecological emergency unit. Patients who opted for open myomectomy as management option after counselling were worked up for the procedure. Those who consented to the study were randomized after considering the inclusion and exclusion criteria. Appropriate investigations including complete blood count, renal and liver function test, and grouping and cross-matching of blood were carried out. Those with medical history of thromboembolic disease, ischemic heart disease, malignancy, liver disease or chronic kidney disease, active or history of thrombotic or thromboembolic disease, and hypersensitivity to tranexamic acid were excluded from the study.

### 4.2. Randomization

Participants were randomized by means of a computer-generated random numbers using the software Research Randomizer®. Thirty-one numbers were randomly generated from a pool of sixty-two numbers and assigned to group A while the remaining numbers were automatically assigned to group B. After assigning the appropriate group to each number, the number was written on each opaque envelope. The envelopes were then arranged in sequential order from 1 to 62.

### 4.3. Drug Administration

Tranexamic acid injection is a plain liquid which contains 500 mg of the drug in 5 ml solution. Two ampoules (10 ml), containing 1 g of the drug, were syringed into a 10 ml syringe (for cases) while 10 ml of water was also syringed into similar 10 ml syringe (for placebo) and packed in corresponding opaque numbered envelopes by the hospital pharmacist. These were sequentially issued to the patients according to the randomization numbers.

Before open myomectomy, the researchers retrieved the labelled envelope containing the unmarked agent (drug or placebo) in a syringe from the pharmacy. The agent was then handed over the anaesthetist who added it into intravenous normal saline (1000 ml) and allowed it to drop at the rate of 100 ml per hour, starting at induction of anaesthesia.

All the patients had tourniquet applied at the isthmus prior to uterine incision, which was released every 45 minutes till the end of the surgery. Intra-abdominal drain was inserted at the end of each procedure, and myoma seedlings were sent for histology. Gravimetric method was used to estimate the blood collected on the abdominal mops, surgical drapes, and surgeon and assistant gowns, using spring balance weighing scale, calibrated in gram, by subtracting their pre-use weight from post-use weight. This was added to the blood collected in the suctioning machine bottle to get the total intraoperative blood loss. Postoperative blood loss was determined by adding the blood collected on wound dressing to that collected inside wound drains. Postoperative pack cell volume was done on second postoperative day using automated blood analyzer.

## 5. Data Analysis

Data collection was done using a predesigned pro forma. Data analysis was carried out using Epi Info software (7.2.1, CDC, Atlanta, Georgia). The results were expressed as frequency tables, percentages, mean, and standard deviation. The relationships between categorical data were analyzed using *X*^2^ and continuous variables with Student's *t*-test. *P* value > 0.05 was considered statistically significant.

Permission for the study was obtained from the Research and Ethical Committee of the Alex Ekwueme Federal University Teaching Hospital, Abakaliki. Clinical trial registration number NCT04560465.

## 6. Results

**Figure 1:**
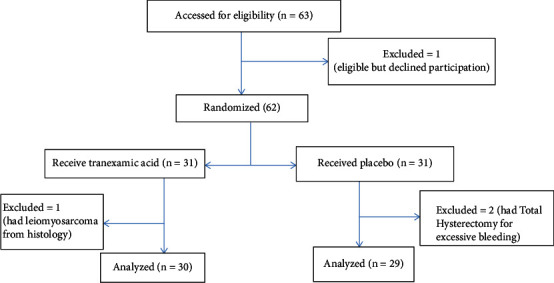
Flow chart of patients through the study.

During the study period, there were 486 gynaecological admissions, out of which 84 were due to symptomatic uterine fibroid (17.3%). Also, there were 296 gynaecological operations of which 63 were myomectomies (21.3%), as shown in the chat flow in Figure 1. Majority of the patients in both groups, in this study, were married, though the proportion of married women (66.7%) in the study group was slightly greater than that of the placebo group (62.1%). Also, majority of the participants were traders (23.7%), housewives (17.0%), and civil servants (17.0%).

Table 1 illustrates the demographic and preoperative clinical characteristics of the participants. And there was no statistically significant difference in demographic characteristics between both groups of study participants. As shown on Table 2, pressure symptoms (59.3%) were the commonest indications for myomectomy, followed by menorrhagia and subfertility. From Table 3, the differences between the type of anaesthesia received, nature of uterine incisions, and surgeons' cadre were not statistically significant. However, the placebo group received significantly more blood transfusion during open myomectomy than the study group.

Table 4 demonstrates the intraoperative and postoperative outcomes. As illustrated, TXA significantly reduced myomectomy-associated blood loss and duration of hospital (*P* value < 0.05). Though there was no significant difference in the number of myomas removed, the operative time was significantly shorter among the participants in the study group compared with the controlled group.

## 7. Discussion

This is a double-blind RCT that determined the efficacy of TXA in reducing blood loss during open-myomectomy surgical procedures. The predesigned pro forma used in this study is in the appendix. The findings of this study demonstrate that tranexamic acid (TXA) significantly reduced the intra- and postoperative, as well as total blood loss associated with open myomectomy in patients with symptomatic uterine fibroids in the cohort of patients recruited. Tranexamic acid also reduced the need for blood transfusion, improved the postoperative haemoglobin concentrations, and reduced the duration of hospital stay in the same participants. This is probably due to the ability of TXA acid to reduce blood loss by inhibiting enzymatic breakdown of fibrins in blood clots through inhibition of conversion of plasminogen to plasmin [15].

In this study, symptomatic uterine fibroid constituted 17.3% of all gynaecological admissions. This was higher than 7% and 8.35% reported in Ilesha and Ile-Ife [16], Southwest Nigeria, respectively; but it is lower than 21.4% from Kano [17], in Northern Nigeria. The higher prevalence in Kano study is a reflection of the fact that not all patients with fibroids are symptomatic [17]. Majority of patients in both groups were either nulliparous or married, which is in keeping with the findings in previous studies [16–23], supporting the fact that fibroid is more common in nulliparous than multiparous women [16–18]. Also, majority of the patients were in their 4^th^ decade of life. This is the peak of reproductive period in women, when reproductive hormones have had adequate interaction with the uterus. This is similar to the findings in other studies [15, 23].

Pressure symptoms were the commonest presenting symptoms in this study. This might be due to late presentation of majority of these cases. This finding was similar to what was reported by Ezeama et al. in Nnewi in which abdominal mass was seen in 67% of cases [23]. This is contrary to menorrhagia being the commonest presenting symptom in Maiduguri [18], possibly because of high prevalence of submucous fibroids in Maiduguri. Subfertility as a presenting symptom was seen mostly in the married patients who were the majority in each group, who possibly presented in search of solution to their fertility problems.

The mean number of fibroid nodules removed was 12.3 for tranexamic acid group and 14.2 for the placebo group. This was greater than 1 to 3 fibroid nodules reported by Saha [5] in India; and this may probably be due to racial differences in fibroid development as black women are at higher risk of multiple fibroid nodules [1]. Also, the mean weights of fibroid nodules removed from both groups of patients were higher than those recorded by Saha [5] and Taylor et al. [19]. This may be due to the fact that most patients with fibroids usually present late in Nigeria, thus allowing the fibroids time to grow to very large size.

This study demonstrates that perioperative tranexamic acid infusion significantly reduces the mean intraoperative and postoperative blood loss when compared to placebo. This supports the effect of the drug on blood loss by promoting clot formation and inhibiting clot dissolution. Baradwan et al., Shaaban et al., and Fusca et al. observed similar findings in their meta-analysis and systemic review of RCTs, where significant reduction of blood loss was recorded following the use of tranexamic acid during myomectomy [14, 20, 24]. However, this differs from the finding in the United States clinical trial where TXA was found to insignificantly reduced blood loss by mean volume of 63 ml, and there was no difference in blood transfusion rate for patients in both arms of the study [13]. The differences in findings might be due to differences in patients' characteristics across the regions where the studies took place.

Also, in this study, tranexamic acid infusion reduced the mean operative time by about 30 minutes, a finding similar to what was reported by Wang et al. [25]. This is because a clear operative field reduces the need to mop blood from the operating field, thus allowing for minimal interruption of the procedure. Similarly, patients who received TXA stayed fewer days in the hospital after surgery compared to the other group. This might be due to fewer requirements for blood transfusion after confirmation of postoperative packed cell volume.

Tranexamic acid significantly reduced blood transfusion requirements in the study group. Accordingly, patients who received TXA had significantly higher postoperative haemoglobin concentrations when compared to the placebo group. This was similar to the observation by Wang et al. [25] that tranexamic acid reduced the need for blood transfusion during myomectomy. The observed ability of tranexamic acid to reduce total blood loss associated with myomectomy, improve postoperative haemoglobin concentration, and reduce the risk of blood transfusion requirements was probably due to its ability to inhibit enzymatic breakdown of fibrins in blood clots by inhibiting the activation of plasminogen to plasmin [15].

This study has demonstrated the efficacy and safety of perioperative 1000 mg TXA in reducing blood loss during open myomectomy, and our findings are similar to those earlier reported. TXA has also been proven to be effective to reduce blood loss in other gynaecological procedures. In a systemic review and meta-analysis of randomized controlled trial, Abu-Zaid et al. reported that TXA is effective in reducing intraoperative blood loss, requirement for postoperative blood transfusion, and requirement for intraoperative topical hemostatic agents among patients undergoing hysterectomy [26]. TXA has also been proven to be effective and safe in reducing postpartum hemorrhage among parturients. Abu-Zaid et al. in a systematic review and meta-analysis of 17 randomized controlled trials reported that prophylactic TXA was linked to decreased incidence rates of postpartum hemorrhage, need for blood transfusion, and need for additional uterotonic agents [27]. Lee et al. also reported that prophylactic TXA can lower PPH occurrence and reduce the need for postpartum blood transfusion in their systemic review and meta-analysis of RCTs [28].

All these studies and systemic reviews showed that prophylactic TXA is safe and effective in reducing blood loss in patients undergoing procedures where major hemorrhages are anticipated and, especially, in patients without background comorbidities that were excluded this study [13–28].

### 7.1. Conclusion

Tranexamic was shown, in this study, to significantly reduce myomectomy-associated blood loss, the requirements for blood transfusion and intraoperative time as well as the duration of admissions.

### 7.2. Strengths of the Study

The study was a double-blind randomized controlled trial. This ensured no bias in patient allocation to either arm of the study. Also, excluding patients with comorbidities helps ensure that there was no unwarranted side effect of TXA among participants.

### 7.3. Limitations of the Study

Surgeon's technique at myomectomy could not be fully standardized due to variation in the sizes and positions of myomas. Also, this is a single centre study in which its findings may not be applicable to other centres.

## 8. Recommendation

Administration of intravenous tranexamic acid intraoperatively to patients undergoing open abdominal myomectomy may limit blood loss during the procedure. Also, prophylactic TXA is safe and effective in reducing blood loss during open myomectomy. However, multicentre studies on this subject are recommended to either corroborate or refute the findings of this study and will help to design a systemic review and meta-analysis of RCTs of efficacy of prophylactic TXA in reducing myomectomy-associated blood loss among this selected group of participants.

## Figures and Tables

**Table 1:** Demographic and baseline clinical characteristics of participants.

Variables	Groups		*t*-test	*P* value
	A (tranexamic acid (*n* = 30))	B (placebo group (*n* = 29))		
	Mean ± SD	Mean ± SD		
Age (years)	35.1 ± 4.9	33.0 ± 6.2	1.45	0.15
Parity	1.8 ± 0.9	1.7 ± 0.8	0.48	0.62
Preoperative haemoglobin	11.0 ± 1.1	11.2 ± 1.4	0.49	0.63
BMI (kg/m^2^)	25.5 ± 1.4	25.9 ± 2.1	0.86	0.39
Preoperative PCV (%)	33.1 ± 3.3	33.6 ± 4.2	0.51	0.61
Bedside clotting time (min)	5.5 ± 1.3	5.4 ± 1.3	0.30	0.77
Platelet count (×10^3^ cell/ml)	168.7 ± 24.8	176.3 ± 28.3	1.10	0.28

BMI = basic metabolic index; PCV = packed cell volume.

**Table 2:** Indications for surgery.

Indication	Number	Percentage (%)
Menorrhagia	Group A = 17	
Yes	Group B = 17	57.6
	Total = 34	

Pressure symptoms	Group A = 18	
Yes	Group B = 17	59.3
	Total = 35	

Subfertility	Group A = 9	
Yes	Group B = 10	32.2
	Total = 19	

**Table 3:** Type of anaesthesia, cadre of surgeons, uterine incision, and blood transfusion.

Variables	Group A *n* = 30 (%)	Group B *n* = 29 (%)	*X* ^2^ value	*P* value
Type of anaesthesia				
General	9 (0.30)	7 (24.1)	0.26	0.61
Spinal	21 (0.70)	22 (75.9)		
Cadre of surgeons			0.29	0.59
Consultant	22 (73.3)	23 (79.3)		
Senior registrar	8 (26.7)	6 (20.7)		
Uterine incisions				
Anterior vertical	20 (66.7)	19 (65.5)	2.36	0.50
Anterior transverse	3 (10.0)	6 (20.7)		
Posterior vertical	6 (20.0)	3 (10.3)		
Posterior transverse	1 (3.3)	2 (6.9)		
Blood transfusion				
Yes	4 (13.3)	16 (55.2)		
No	26 (86.7)	13 (44.8)	11.52	0.002

**Table 4:** The intraoperative and postoperative parameters of both groups.

Continuous variables	Groups		*t* value	95% CI	SMD	*P* value
	Tranexamic acid group (A) (*n* = 30)	Placebo group (B) (*n* = 29)				
	Mean (SD)	Mean (SD)				
Intra-op blood loss (ml)	413.6 (165.6)	713.6 (236.3)	5.66	-403.9 to -196.1	-0.212	<0.0001
Intra-op time (min)	89.0 (28.9)	120.0 (23.1)	4.54	-44.7 to -17.7	-3.301	<0.0001
Total number of fibroid nodules removed	12.3 ± 10.8	14.2 ± 9.6	0.71	-3.5 to 7.3	1.900	0.48
Total weight of fibroid removed	1.1 ± 0.5	1.1 ± 0.9	0.00	-0.4 to 0.4	0.000	1.00
Post-op blood loss (ml)	70.0 (0.0)	236.7 (61.8)	14.78	-189.3 to -144.1	-3.811	<0.0001
Post-op stay (days)	5.8 (2.1)	7.8 (1.9)	3.83	-3.1 to -1.0	-3.929	0.0003
Post operation HB (g/dl)	9.7 (1.0)	7.8 (1.5)	5.74	1.2 to 2.6	-0.122	<0.0001
Total blood loss (ml)	474.8 (141.0)	1007.1 (358.5)	7.55	-670.4 to -391.2	30.622	<0.0001

CI = confidential interval; SMD = standardized mean difference.

## Data Availability

Data used in this study can be accessed by direct permission of the authors. Request can be sent to ayodele_olaleye@yahoo.com.

## Appendix

### Efficacy of Tranexamic Acid in Reducing Myomectomy Associated Blood Loss in FETHA

#### A.1. Questionnaire

Instruction: please tick as appropriate as any information given would be treated with utmost confidentiality.

**SECTION A: SOCIO-DEMOGRAPHIC DATA**

1. Age; 20-24( ) 25-29( ) 30-34( ) 35- 39( ) ≥40 ( )
2. Marital Status; Single ( ) Married( ) Separated( ) Divorced( )
3. Parity; 0 ( ) 1-4 ( ) 5-9 ( ) ≥10 ( )
4. Religion; Islam( ) Catholic( ) Anglican( ) Protestant ( )Pentecostals( ) Others( )
5. Occupation; Health worker( ), Teacher( ), Banker( ), Lecturer( ), Other Civil Servants( ), Trading( ), Artisan ( ), Farming ( ) House wife ( ) students ( )
6. Height (cm); <150 ( ) 150-159 ( ) 160-169( ) 170-179 ( ) 180-189 ( ) ≥190 ( )
7. Weight (kg); <50 ( ) 50-59 ( ) 60-69 ( ), 70-79 ( ) 80-89 ( ) 90-99 ( ) ≥100 ( )

**SECTION B: INDICATION FOR SURGERY AND PREOPERATIVE PARAMETERS**

(8) Indication for surgery; Menorrhagia Yes ( ) No ( ), Pressure Symptoms Yes( ) No ( ), Subfertility Yes ( ), No ( ), Others …………..
(9) Level of Surgeon; Consultant ( ), Senior Registrar ( )
(10) Preoperative PCV (%); <30 ( ) 30-34 ( ) 35-39 ( ) ≥ 40 ( )
(11) Bedside Clotting Time (min); ≤2 ( ) 3-8 ( ) > 8 ( )
(12) Absolute platelets Counts; ≤150 ( ), 151-200 ( ), 201-250 ( ), 251-300 ( ), 301-350 ( ), 351-400 ( ), >400 ( )

**SECTION C: INTRAOPERATIVE PARAMETERS**

(13) Type of uterine incision; Anterior Vertical ( ), Anterior Transverse ( ), Bonney's Hood ( ), Posterior Vertical ( ), Posterior Transverse ( ),
(14) Number of fibroid nodules; 1-10 ( ), 11-20 ( ), 21-30 ( ), 31-40 ( ),41-50 ( ), ≥50 ( )
(15) Volume of biggest fibroid nodule (cm^3^); 1-10 ( ), 11-20 ( ), 21-30 ( ), 31-40 ( ), 41-50 ( ), ≥50 ( )
(16) Weight of fibroids (kg); <1( ), 1-5 ( ), 6-10 ( ), 11-15 ( ), 16-20 ( ), >20 ( )
(17) Volume of smallest fibroid nodule (cm^3^); <1 ( ), 1-5 ( ), 6-10 ( ), 11-15 ( ), 16-20 ( ), >20 ( )
(18) Intraoperative blood loss (mls); <150 ( ), 150-290 ( ), 300-440 ( ), 450-590 ( ), 600-740 ( ), 750-890 ( ), 900-1040 ( ), ≥1050 ( )
(19) Intraoperative blood transfusion Yes ( ), No ( ). If yes how many pints 1-2 ( ), 3-4 ( ), ≥5 ( )
(20) Operative time (minutes); <60 ( ), 60-89 ( ), 90-119 ( ), 120-149 ( ), 150-179 ( ). ≥180 ( )

**SECTION D: POSTOPERATIVE PARAMETRES**

(21) Any postoperative bleeding; Yes ( ), No ( )
(22) If Yes, what volume (mls):<150 ( ), 159-290 ( ), 300-440 ( ),450-590 ( ), 600-740 ( ), ≥750 ( )
(23) Postoperative PCV (%); <20 ( ), 20-24 ( ), 25-29 ( ), 30 34 ( ), 35-39 ( ). ≥40 ( )
(24) Postoperative blood transfusion; Yes ( ), No ( )
(25) If Yes, number of pint(s): 1-2 ( ), 3-4 ( ), ≥5 ( )
(26) Duration of hospital stay (days); <5 ( ), 5-9 ( ), 10-15 ( ), ≥15 ( )
(27) Total blood loss (mls); <500 ( ), 500-740 ( ), 750-990 ( ), 1000-1240 ( ), 1250-1490 ( ), ≥1500 ( )
